# Comparison of rapid direct in vitro antimicrobial susceptibility testing and agar disk diffusion for bovine mastitis pathogens

**DOI:** 10.3168/jdsc.2026-1073

**Published:** 2026-06-25

**Authors:** Breno Luis Nery Garcia, Carlos Eduardo Fidelis, Kristian da Silva Barbosa, Gustavo Freu, Marcos Veiga dos Santos

**Affiliations:** 1 Faculty of Veterinary Medicine, University of Calgary, T2N 1N4, Calgary, AB, Canada; 2 Department of Animal Nutrition and Production, School of Veterinary Medicine and Animal Science, University of São Paulo, Pirassununga, 13635-900, São Paulo, Brazil

## Abstract

The rise in antimicrobial resistance (AMR) represents a global challenge for both animal production and public health and is partly driven by the overuse of antimicrobials in animal production. On dairy farms, the treatment of clinical mastitis (CM) is a major contributor to antimicrobial use, making its judicious use in CM management critical for public health. Currently, in some dairy herds, antimicrobial selection for CM is often made without susceptibility testing because of logistical challenges associated with laboratory testing. Rapid and direct in vitro antimicrobial susceptibility tests (RDIAST) could help guide treatment and reduce the risk of development of AMR in dairy herds. This study evaluated the agreement between RDIAST and standard agar disk diffusion for CM isolates. A total of 70 *Staphylococcus aureus*, 69 *Staphylococcus chromogenes*, 70 *Streptococcus uberis*, 70 *Klebsiella* spp., and 69 *Escherichia coli* isolates from a mastitis pathogen library were randomly selected. Susceptibility was assessed using both methods, and agreement was measured using Cohen's kappa, overall agreement, Bland–Altman (BA) analysis of inhibition zones, sensitivity (Se), and specificity (Sp). Overall agreement ranged from 0.73 (*Klebsiella* spp.) to 0.89 (*S. chromogenes*), whereas kappa values ranged from 0.09 (*S. chromogenes*) to 0.47 (*Klebsiella* spp.), indicating fair to moderate agreement. The BA analysis showed differences in inhibition zones ranging from −7.42 mm (*S. chromogenes*) to 0.68 mm (*S. uberis*), with a general bias of −2.81 mm, suggesting that the inhibition zones were generally smaller with RDIAST, and overall Se and Sp were both 0.74. These results underscore the importance of considering species-specific differences in inhibition zones when interpreting RDIAST results. Further validation is needed before RDIAST can be reliably implemented for on-farm antimicrobial selection in CM treatment.

## Graphical Abstract

**Figure:**
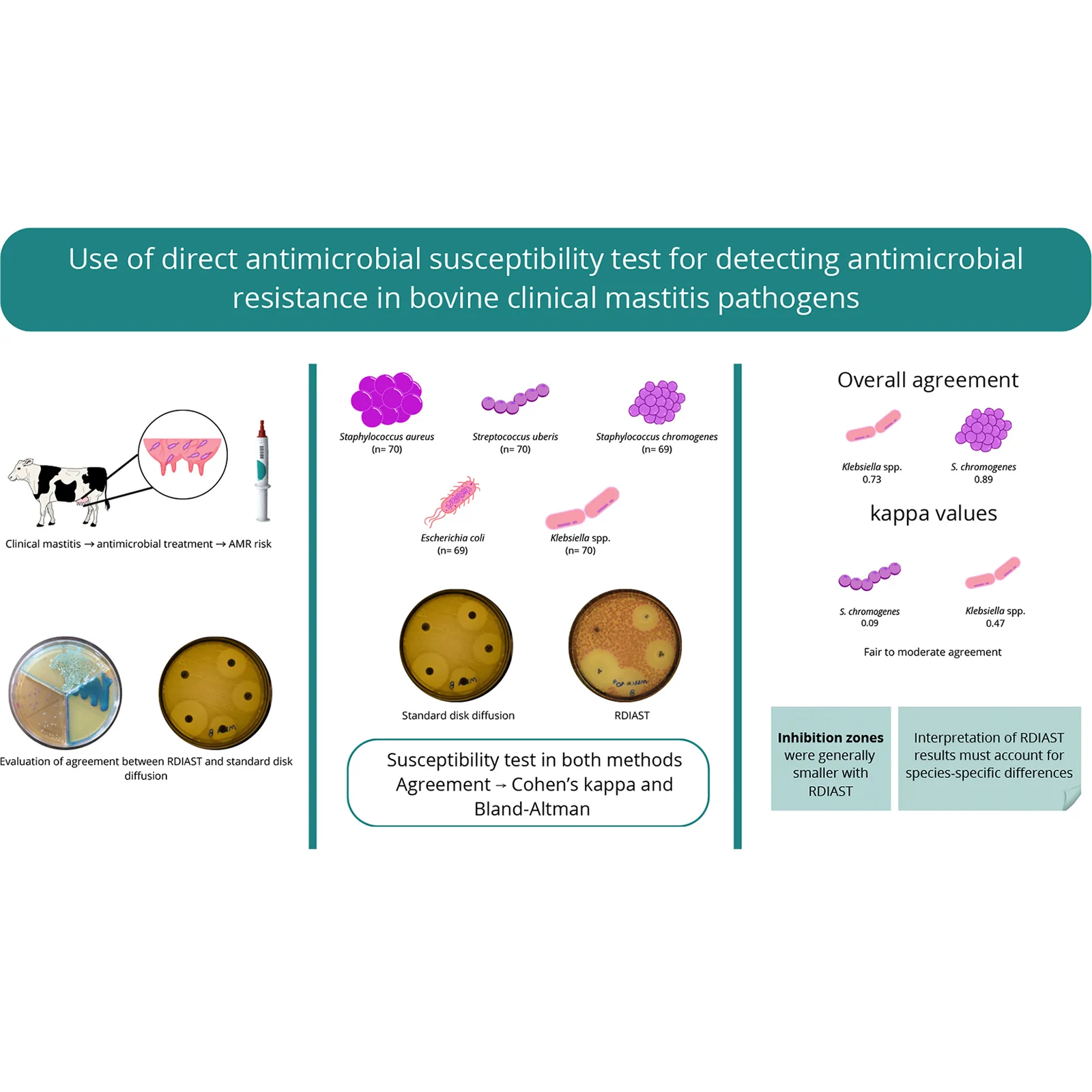

Mastitis is the most prevalent disease affecting dairy cattle and the leading cause of antimicrobial use (**AMU**) on dairy farms (Saini et al., 2013; Tomazi and dos Santos, 2020; de Jong et al., 2023). Although the specific contribution of AMU for mastitis treatment to the transmission of antimicrobial resistance (**AMR**) from livestock to humans remains difficult to quantify, substantial evidence supports the role of livestock AMU in the emergence and dissemination of AMR, raising societal and public health concerns (Tang et al., 2017). This concern has been emphasized by the World Health Organization's Global Action Plan on AMR (FAO OIE WHO, 2003). These factors highlight the need for strategies that promote judicious AMU in the management of clinical mastitis (**CM**). From a public health perspective, implementing approaches that ensure rational AMU in CM treatment is essential.

The adoption of rapid microbiological identification methods for the selective treatment of CM (Lago et al., 2011; Lago and Godden, 2018; Granja et al., 2021), alongside tools that enable informed antimicrobial selection, is critical to avoid the unnecessary use of antimicrobials that are essential in human medicine and often unnecessary for managing mastitis (Nobrega et al., 2020). Although some countries have regulations limiting the use of these drugs (Simjee and Ippolito, 2022), in others, such as Brazil, they are still routinely employed (Tomazi and dos Santos, 2020), potentially facilitating the development and spread of AMR (WHO, 2005).

Ongoing surveillance of the antimicrobial susceptibility of mastitis pathogens using methods such as agar disk diffusion can support more rational antimicrobial selection by revealing pathogen-specific resistance patterns (de Jong et al., 2018). However, logistical challenges, such as the need to send milk samples to laboratories and the prolonged time required to obtain results, limit the feasibility of these tests for on-farm decision-making regarding CM treatment in dairy cows. Because delaying treatment decisions for CM while awaiting antimicrobial susceptibility test (**AST**) results is impractical, as processing and shipping can take 4 to 5 d, alternative strategies are necessary.

Rapid and direct in vitro antimicrobial susceptibility tests (**RDIAST**) could be an alternative approach for testing the susceptibility of microorganisms to antimicrobials through the direct inoculation of biological samples (e.g., milk) or isolated colonies into appropriate culture media. This approach eliminates the need for laboratory-based testing and provides rapid results within approximately 24 to 26 h (Coorevits et al., 2015; Arienzo et al., 2023). However, RDIAST has limitations, including a lack of control over the microbial load in the inoculum and the occurrence of mixed cultures (i.e., more than one microorganism being present in the same sample), which requires careful interpretation of the results.

Despite these limitations, because decisions regarding CM treatment are currently made without AST results (El Garch et al., 2020), RDIAST may offer an alternative to improve antimicrobial selection for CM treatment protocols. Although a clear association between bacteriological cure outcomes in CM and AST results of mastitis-causing pathogen has not been observed in previous studies using conventional laboratory techniques (Hoe and Ruegg, 2005; Apparao et al., 2009a,b), the use of innovative and rapid AST methods that can be performed on farm may provide a practical solution. Selecting antimicrobials based on RDIAST results could contribute to promoting rational AMU in dairy herds.

Although previous studies have evaluated field-based diagnostic tests for mastitis (Mansion-de Vries et al., 2014; Jones et al., 2019), to our knowledge, this is the first study to assess the agreement between an on-farm–suitable RDIAST and standard methodologies (agar disk diffusion) for bacterial isolates from CM. The experimental protocols were carried out in accordance with the ethical principles of animal experimentation and were approved by the Ethics Committee on the Use of Animals (CEUA) of the School of Veterinary Medicine and Animals Sciences of the University of São Paulo (SVMAS-USP), under registration CEUA 7544101221.

Bacterial isolates were obtained from the mastitis pathogen library of the Milk Quality Research Laboratory (Qualileite; School of Veterinary Medicine and Animal Science, University of São Paulo, Pirassununga, Brazil), which comprises 4,870 isolates distributed across 139 species. These isolates originated from milk samples collected from cows with CM (n = 1,221) and subclinical mastitis (n = 3,649) from 102 dairy herds in Brazil. Pure cultures of the bacterial isolates were previously identified using MALDI-TOF, as described by Barcelos et al. (2019), and stored at −80°C. From this library, isolates from CM cases were randomly selected to represent the following species: *Staphylococcus aureus* (n = 70), *Streptococcus uberis* (n = 70), and *Klebsiella* spp. (*Klebsiella pneumoniae*, n = 53; *Klebsiella oxytoca*, n = 4; *Klebsiella variicola*, n = 1), *Escherichia coli* (n = 69), and *Staphylococcus chromogenes* (n = 69). To determine the total number of bacterial isolates required for evaluation, a 15% difference was estimated between the antimicrobial susceptibility results obtained using the 2 methodologies (rapid and standard). Using a type I error of 0.05 and type II error of 0.2, the calculated sample size was 69 isolates (Sealed Envelope Ltd., 2012).

The selected isolates were simultaneously subjected to 2 methodologies for in vitro antimicrobial susceptibility testing: (1) the standard (agar disk diffusion, performed according to the guidelines of CLSI, 2025) and (2) RDIAST.

Cryopreserved isolates, stored in 2 mL of brain heart infusion broth, were inoculated onto blood agar plates containing 5% bovine blood (**BA**) and incubated at 37°C for 24 h. After incubation on BA, the isolates were prepared for each of the 2 testing methodologies as follows:

For the agar disk diffusion method, colonies (1 to 3) of each isolate were transferred from BA to test tubes containing 5 mL of sterile saline solution using a platinum loop. The suspensions were homogenized using a vortex mixer and standardized to 0.5 McFarland (∼10^8^ cfu/mL) using a turbidimeter (Uniscience, São Paulo, Brazil). The suspension was then evenly inoculated onto Mueller–Hinton agar (**MHA**) plates using a sterile swab.

For the RDIAST methodology, chromogenic medium, Mueller–Hinton Orientation (**MHO**, CHROMagar, France), was used. Mueller–Hinton Orientation is a nonselective culture medium intended for antimicrobial susceptibility testing and facilitates presumptive microbial identification through the visual interpretation of colony color patterns, according to the manufacturer's guidelines. Because the RDIAST methodology was designed for on-farm use, bacterial colonies were directly inoculated from the BA plates onto the MHO medium without prior inoculum standardization. A sterile swab was used to evenly transfer the colonies across the entire surface of chromogenic medium agar.

After inoculating the MHA and MHO plates, disks containing the following antimicrobials were placed onto the plates: enrofloxacin (5 µg), kanamycin (30 µg), penicillin-novobiocin (10 µg), oxacillin (30 µg), ceftiofur (30 µg), gentamicin (10 µg), neomycin (30 µg), ampicillin (10 µg), clindamycin (2 µg), cephalexin (30 µg), amoxicillin + clavulanate (30 µg), and tetracycline (1 µg). Considering that the objective was to develop a rapid test suitable for on-farm use, the antimicrobial disks were standardized to ensure consistent assessment of susceptibility for the most frequently used intramammary antimicrobial formulations available in Brazil (Tomazi and dos Santos, 2020; Martins et al., 2021; Piaia et al., 2025), regardless of the pathogen species.

To ensure proper disk placement, 2 MHO and 2 MHA plates were prepared for each isolate, with 6 disks placed equidistantly on every plate. A template was developed to ensure accurate disk positioning and avoid errors in the order and placement of disks on the plates.

The plates were incubated at 37°C for 24 h. Following incubation, visual inspection of colony coloration on the MHO plates was performed, and the inhibition zone diameters were measured on both MHO and MHA plates. The inhibition zone diameters were measured using an automated colony counter with Scan Interscience software (Scan 1200; Interscience, Paris, France). The results were recorded in a Microsoft Excel (Microsoft Corp., Redmond, WA) spreadsheet, and the plates were photographed for documentation. Photographs were captured directly using the colony counter's “photograph” function of the Scan Interscience software (Figure 1). All antimicrobial susceptibility tests were performed in accordance with the guidelines of CLSI (2025) and EUCAST (2025), using *Staphylococcus aureus* ATCC 29213 and *Escherichia coli* ATCC 25922 as quality control strains. If the quality control strains presented unexpected results, the analysis was repeated to ensure accuracy and reproducibility of the results.

**Figure 1:**
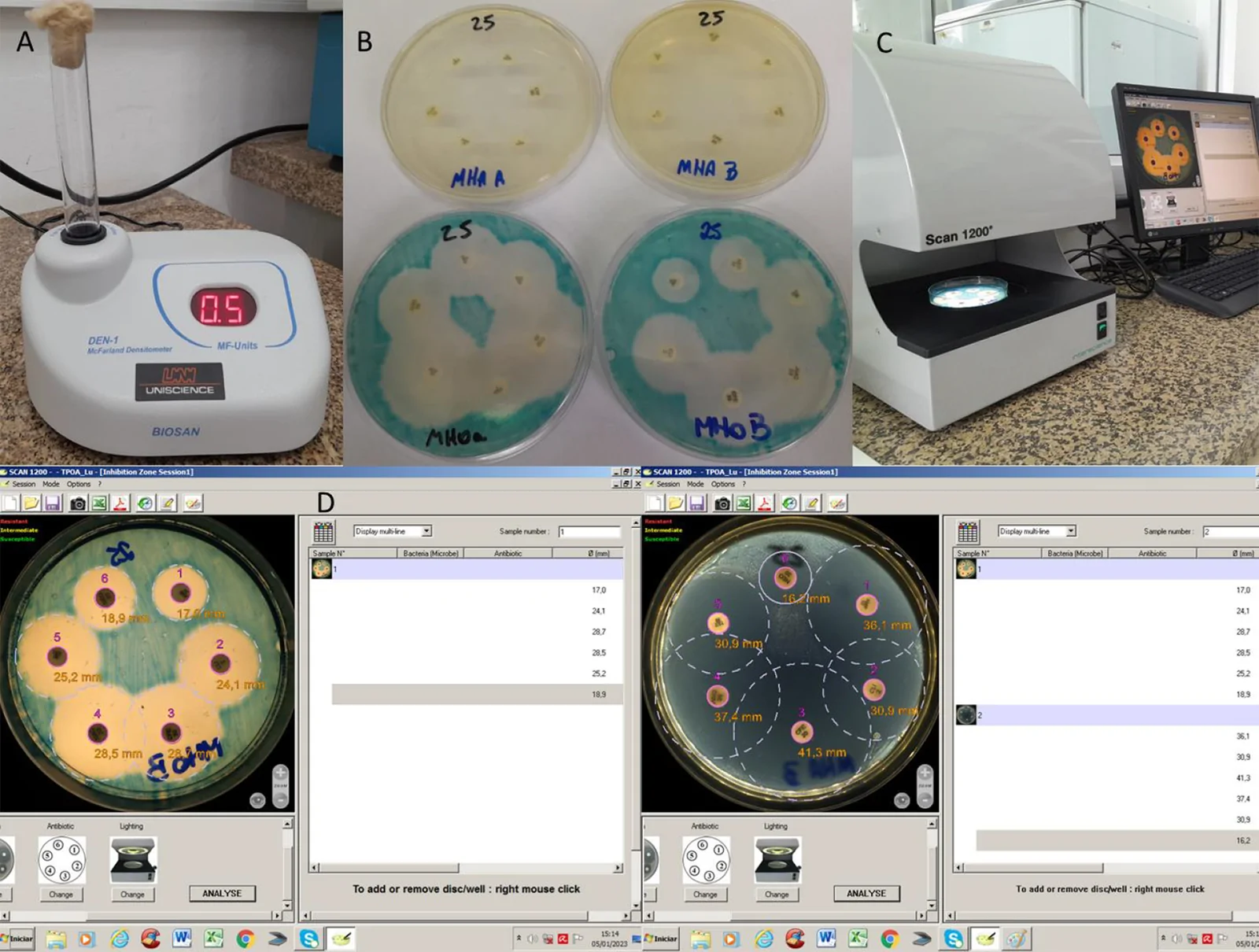
In vitro antimicrobial susceptibility testing by agar disk diffusion and rapid and direct in vitro antimicrobial susceptibility tests (RDIAST): (A) turbidimeter for standardizing the bacterial suspension to 0.5 McFarland; (B) MHO and MHA plates after incubation at 37°C for 24 h, ready for reading; (C) automated colony counter; and (D) measurement of inhibition zones (mm).

The inhibition zone diameters measured on both MHA and MHO plates were interpreted according to CLSI (2024, 2025) and EUCAST (2025) breakpoints. Based on the inhibition zone diameters expressed in millimeters, the isolates were classified as susceptible (**S**), intermediate (**I**), or resistant (**R**). Because binary outcomes were required for comparison and agreement assessment between methodologies, both S and I results were coded as 0, whereas R was coded as 1 for analysis. The mean inhibition zone diameter and the proportions of S and R isolates for each antimicrobial tested on each culture medium are presented in Supplemental Tables S1 to S5 (see Notes).

Clinical breakpoints were applied in accordance with the CLSI VET01S and EUCAST version 15.0. veterinary guidelines whenever available. In cases where no breakpoint was defined for a particular species, the corresponding human (CLSI, 2024) breakpoint was used. For antimicrobials without a species-specific breakpoint, the interpretive criteria of an alternative agent within the same antimicrobial class were applied. These adaptations were necessary because of the limited availability of validated breakpoints for mastitis isolates (Hoe and Ruegg, 2005).

For species with no established interpretative guidelines, isolates were grouped into 4 mastitis-causing bacterial categories for breakpoint interpretation: (1) *Staphylococcus* spp., (2) *Streptococcus* spp., (3) *Enterococcus* spp., and (4) *Enterobacteriales*. *Lactococcus* spp. and *Aerococcus* spp. are phylogenetically related to *Streptococcus* spp. within the order *Lactobacillales*; therefore, antimicrobial susceptibility was interpreted using *Streptococcus* spp. breakpoints (Humphries and Hindler, 2014; Kolar et al., 2024).

The agreement between the methodologies was calculated using Cohen's kappa coefficient (*k*) according to the following formula:

$$k=\frac{p_{0}-p_{e}}{1-p_{e}},$$

where *k* is Cohen's kappa coefficient, *p*_0_ is observed agreement, and *p_e_* is the hypothetical probability agreement.

The *k* results were categorized according to the level of agreement as follows: almost perfect (0.81 to 1.00), substantial (0.61 to 0.80), moderate (0.41 to 0.60), fair (0.21 to 0.40), slight (0.00 to 0.20), and poor (<0.00; Landis and Koch, 1977). The agreement between the inhibition zone diameters obtained by each method was evaluated using Bland–Altman analysis. The overall agreement was calculated as the ratio of concordant results between the 2 methods to the total number of measurements. Additionally, the sensitivity (**Se**) and specificity (**Sp**) of the RDIAST were assessed using the standard agar disk diffusion method as the gold standard to determine true positives, true negatives, false positives, and false negatives for the binary outcome of AMR for each antimicrobial–species combination. R software (version 4.1.3; https://www.r-project.org/) was used to calculate the diagnostic performance indicators. The *irr* package was used to calculate *k* and overall agreement, the *bdpv* package was used to estimate Se and Sp, and the *blandr* package was used for Bland–Altman analysis.

The agreement between RDIAST and the standard methodology varied according to the pathogen species, ranging from 0.73 for *Klebsiella* spp. to 0.89 for *Staphylococcus chromogenes*, while the *k* values ranged from 0.09 for *Staphylococcus chromogenes* to 0.47 for *Klebsiella* spp. (Table 1).

**Table 1:** Cohen's kappa coefficient (*k*), Bland–Altman agreement analysis, sensitivity (Se), specificity (Sp), and overall agreement between rapid and direct in vitro antimicrobial susceptibility tests and the standard agar disk diffusion method in bacterial isolates from clinical mastitis cases (n = 348)

Species	*k*	*P*-value	Agreement	BA^1^ (95% CI)	Se (95% CI)	Sp (95% CI)
Overall^2^ (n = 348)	0.41	<0.001	0.8	−2.81	0.74	0.74
				(−3.03 to −2.59)	(0.72–0.77)	(0.72–0.75)
*Staphylococcus aureus* (n = 70)	0.41	<0.001	0.77	−1.95	0.72	0.77
				(−2.21 to −1.68)	(0.69–0.76)	(0.76–0.79)
*Staphylococcus chromogenes* (n = 69)	0.09	0.12	0.89	−7.42	0.89	0.60
				(−7.76 to −7.09)	(0.85–0.92)	(0.55–0.65)
*Streptococcus uberis* (n = 70)	0.32	<0.001	0.77	0.68	0.36	0.83
				(0.16 to 1.20)	(0.29–0.45)	(0.80–0.87)
*Escherichia coli* (n = 69)	0.4	<0.001	0.84	−1.64	0.72	0.85
				(−1.94 to −1.34)	(0.62–0.83)	(0.82–0.88)
*Klebsiella* spp. (n = 70)	0.47	<0.001	0.73	−0.83	0.89	0.60
				(−1.48 to −0.17)	(0.85–0.93)	(0.55–0.65)

1 Mean difference bias in the Bland–Altman agreement analysis.

2 Overall results for all pathogen species evaluated.

Considering the interpretative categories of *k* values, for all evaluated pathogen species, RDIAST demonstrated agreement levels ranging from slight (0.00 to 0.20) to moderate (0.41 to 0.60) with the standard methodology (Landis and Koch, 1977). The mean difference (bias) in the inhibition zone diameters, evaluated through Bland–Altman agreement analysis, ranged from −7.42 mm for *Staphylococcus chromogenes* to 0.68 mm for *Streptococcus uberis*, with an overall bias of −2.81 mm. These results indicate that the inhibition zone diameters tended to be smaller in RDIAST than in the standard methodology. The only pathogen for which the inhibition zones were larger in RDIAST was *Streptococcus uberis*, for which a positive bias of 0.68 mm was observed.

This limited agreement may be attributed to the lack of standardization in the microbial inoculum to 0.5 McFarland (approximately 10^8^ cfu/mL), as required by the antimicrobial susceptibility testing guidelines for standard methodology (CLSI, 2025). This variation was further underscored by the overall bias of −2.81 mm observed in the inhibition zone diameter observed in the RDIAST, which may be related to the higher concentration resulting from the direct inoculation of colonies onto the chromogenic culture plate. This pattern was observed across all species (ranging from −0.83 to −7.42 mm), except for *Streptococcus uberis*, in which a positive bias of 0.68 mm was observed in the inhibition zone diameter. This exception may be associated with the smaller colony size typical of the *Streptococcus* spp. group (NMC, 2017), potentially resulting in a lower bacterial yield when colonies were collected from BA plates. Although this represents a major limitation of the study, the RDIAST method was specifically designed for on-farm application, where access to laboratory equipment such as turbidimeters is limited or unavailable, relying instead on bacterial growth recovered directly from OFC plates. Therefore, strict inoculum standardization would likely reduce the practical applicability of the method under field conditions, which was the main gap this approach was intended to address.

The overall Se and Sp of the method were both 0.74. Sensitivity ranged from 0.36 (*Streptococcus uberis*) to 0.89 (*Klebsiella* spp. and *Staphylococcus chromogenes*), whereas Sp ranged from 0.60 (*Klebsiella* spp. and *Staphylococcus chromogenes*) to 0.82 (*Escherichia coli*). With the exception of *Streptococcus uberis*, for which the low performance may be associated with the positive bias observed in inhibition zone diameters, all Se estimates were above 0.70, indicating reasonable potential for use as a screening tool to identify AMR in mastitis pathogens. However, the Sp results indicate that approximately 26% of isolates were incorrectly classified as resistant, which could lead to inappropriate treatment decisions due to false-positive results. In practice, this misclassification could promote unnecessary use of broader-spectrum or critically important antimicrobials.

Although no other studies have reported the use of RDIAST for antimicrobial susceptibility testing of CM isolates under field conditions, a simplified agar disk diffusion method has been tested for validation in clinical laboratory settings for mastitis isolates (Kurumisawa et al., 2021). Like our RDIAST methodology, this simplified approach involved the direct inoculation of colonies using a swab, which resulted in smaller inhibition zone diameters. Additionally, another RDIAST methodology was evaluated in the context of urinary tract infections in companion animals (Guardabassi et al., 2015). In that study, the tested RDIAST was reported to have an 83% Se and 100% Sp compared with the standard methodology (MIC), with correct interpretation of susceptibility results reaching 76% when interpreted by experts. However, the *k* results assessing the agreement between RDIAST and the respective standard methodologies (agar disk diffusion and MIC) were not reported in either study, limiting direct comparisons. Although Se is an important metric for evaluating the accuracy of the tested methodology, reflecting its ability to correctly identify resistant pathogens, agreement with the standard methodology remains a critical feature, particularly when considering RDIAST as a tool to promote judicious AMU in treatment selection.

While the RDIAST was designed as a direct on-farm testing approach, in the present study it was performed using bacterial isolates recovered from a cryopreserved mastitis isolate library (−80°C storage) originally obtained from CM cases. This represents a limitation of the study and highlights the need for future investigations evaluating the performance of the method under field conditions. Moreover, the level of agreement observed between the evaluated RDIAST methodology and the standard agar disk diffusion method may limit its applicability as a routine tool for on-farm decision-making regarding CM antimicrobial treatment protocols. However, disregarding the direct interpretation of antimicrobial susceptibility outcomes and instead focusing on RDIAST as a proxy indicator to guide specific antimicrobial avoidance, there may be opportunities for further development of the RDIAST. As machine learning algorithms have already been applied to image interpretation of on-farm microbiological culture results (Garcia et al., 2024), it is possible that similar adaptations could be developed to interpret the inhibition zone sizes on chromogenic culture media plates. Nonetheless, the observed bias could result in false-positive resistance classifications, potentially encouraging the use of clinically important antimicrobials in humans, which are generally unnecessary for CM therapy (Nobrega et al., 2020). Considering this, we cannot recommend RDIAST for on-farm antimicrobial treatment selection.
